# Genome-wide identification of bZIP transcription factors and their expression analysis in *Platycodon grandiflorus* under abiotic stress

**DOI:** 10.3389/fpls.2024.1403220

**Published:** 2024-05-28

**Authors:** Zhen Wang, Panpan Wang, Huiyan Cao, Meiqi Liu, Lingyang Kong, Honggang Wang, Weichao Ren, Qifeng Fu, Wei Ma

**Affiliations:** ^1^ Pharmacy of College, Heilongjiang University of Chinese Medicine, Harbin, China; ^2^ Research Office of Development and Utilization of Medicinal Plants, Heilongjiang Academy of Forestry, Yichun, China; ^3^ Experimental Teaching and Practical Training Center, Heilongjiang University of Chinese Medicine, Harbin, China

**Keywords:** *Platycodon grandiflorus*, bZIP transcription factor, evolutionary analyses, expression profiling, abiotic stress

## Abstract

The Basic Leucine Zipper (bZIP) transcription factors (TFs) family is among of the largest and most diverse gene families found in plant species, and members of the bZIP TFs family perform important functions in plant developmental processes and stress response. To date, *bZIP* genes in *Platycodon grandiflorus* have not been characterized. In this work, a number of 47 *PgbZIP* genes were identified from the genome of *P. grandiflorus*, divided into 11 subfamilies. The distribution of these *PgbZIP* genes on the chromosome and gene replication events were analyzed. The motif, gene structure, *cis*-elements, and collinearity relationships of the *PgbZIP* genes were simultaneously analyzed. In addition, gene expression pattern analysis identified ten candidate genes involved in the developmental process of different tissue parts of *P. grandiflorus*. Among them, Four genes (*PgbZIP5*, *PgbZIP21*, *PgbZIP25* and *PgbZIP28*) responded to drought and salt stress, which may have potential biological roles in *P. grandiflorus* development under salt and drought stress. Four hub genes (*PgbZIP13*, *PgbZIP30*, *PgbZIP32* and *PgbZIP45*) mined in correlation network analysis, suggesting that these *PgbZIP* genes may form a regulatory network with other transcription factors to participate in regulating the growth and development of *P. grandiflorus*. This study provides new insights regarding the understanding of the comprehensive characterization of the PgbZIP TFs for further exploration of the functions of growth and developmental regulation in *P. grandiflorus* and the mechanisms for coping with abiotic stress response.

## 1 Introduction

*Platycodon grandiflorus* (Jacq.) A. DC. is a perennial herbal in the *Platycodon* genus of the Campanulaceae family (Ma et al., 2016). Mainly from the northeastern, northern, central and eastern provinces of China, it is also found in the Russian Far East and the Korean Peninsula (Zhang et al., 2015; Zhang et al., 2015). *P. grandiflors* has high medicinal and edible value, and is a homologous category of medicine and food. At the same time, it also has high ornamental value (Ji et al., 2020). *P. grandiflorus* is rich in natural chemical products. In the past many years, at least 100 compounds have been isolated, including steroidal saponins, sterols, flavonoids and phenolic acids. Modern pharmacological studies have shown that the pharmacological components of *P. grandiflorus* are triterpenoid saponins, and Platycodon D possesses several medicinal activities such as anti-obesity, anti-inflammatory, antioxidant, antitumor and immune regulatory activities (Zhang et al., 2022; Zhang et al., 2022). The growth and development of *P. grandiflorus* is a delicate process, and the study of molecular mechanisms of resistance to abiotic stress is an important production guide in the face of increasingly challenging natural environments. Transcription factors (TFs) are key regulatory proteins with DNA binding domains that can inhibit or activate gene expression (Tian et al., 2023). The YABBY (Kong et al., 2023), WRKY (Jing et al., 2022) and Trihelix (Liu et al., 2023) TF families have been proven to play a role in the response of *P. grandiflorus* to abiotic stress. However, this is only a drop in the ocean of a complex transcriptional regulatory network.

The Basic Leucine Zipper (bZIP) TFs are among of the most widely families of TFs in eukaryotes (Zhao et al., 2016b). The plant bZIP protein has two highly conserved domains composed of 60–80 amino acids: the basic region and leucine zipper region (Nijhawan et al., 2008). bZIP transcription factors mainly regulate tissue and organ development in plants, including seed germination and maturation, embryonic development, flowering, and light morphogenesis (Gangappa and Botto, 2016). Earlier research has indicated that the *bZIP* gene family *ABF1* gene in *Arabidopsis thaliana* regulate seed dormancy and germination, affecting winter *A. thaliana* growth (Sharma et al., 2011). *Litchi chinensis LcbZIP1*, *LcbZIP4*, *LcbZIP7*, *LcbZIP21* and *LcbZIP28* may be involved in the regulation of senescence during postharvest storage of fruit (Hou et al., 2022). Meanwhile, the *bZIP* family also performs essential functions in responding to abiotic stress and regulating secondary metabolites. Overexpression of *Capsicum annuum CabZIP25* in *A. thaliana* can improve tolerance to salt stress (Gai et al., 2020). Similarly, *TabZIP15* can also improve salt tolerance in *Triticum aestivum* (Bi et al., 2021). Overexpression of *Phyllostachys edulis PhebZIP47* in *A. thaliana* and *Oryza sativa* can increase the drought resistance of plants at the adult stages (Lan et al., 2023). In transgenic *Artemisia annua* overexpressing *AabZIP9*, the biosynthetic accumulation of artemisinin, dihydroartemisinic acid, and artemisinic acid was significantly increased (Shen et al., 2019). Reports on *bZIP* genes have now been carried out in a wide variety plants, However, it is unclear whether *bZIP* genes are participating in the growth process of *P. grandiflorus* and their role in responding to abiotic stress. At present, the chromosome-scale genome of *P. grandiflorus* has been released (Lee et al., 2023). Therefore, explore the possible role of bZIP TFs in the growth, development, and abiotic stress of *P. grandiflorus* on the basis of genome-wide and transcriptome data is of great significance.

In this study, we carried out a genome-wide identification and characterization of the *bZIP* gene family of *P. grandiflorus* and comprehensively analyzed the physicochemical properties, phylogeny, synteny relationship, gene structure and *cis*-elements. In addition, by mine the expression patterns of *PgbZIP* genes in eight tissues and constructing correlation networks, it was determined that they may interact with different genes and participate in multiple biological processes together. More importantly, the expression of *PgbZIP* genes during abiotic stress was analyzed by RT-qPCR, which indicated that some *PgbZIP* genes were responsive to salt and drought stress. The present study was conducted to provide a reference for the screening of *bZIP* candidate genes involved in regulating the developmental process of *P. grandiflorus* and in resistance to drought and salinity stress.

## 2 Materials and methods

### 2.1 Plant materials

The plant material *P. grandiflorus* was cultivated from the Medicinal Botanical Garden of Heilongjiang University of Chinese Medicine (HLJUCM), Harbin, China. There are two types of plant materials used in this study, one of which was the roots, stems, leaves and flowers of 1-year-old *P. grandiflorus* in the normal growth state without treatment. The other was a three-month-old *P. grandiflorus* seedling grown in a simulated abiotic stress environment. *P. grandiflorus* seedlings were watered with 200 mmol/L NaCl and 20% PEG6000 (Coolaber Science & Technology, Beijing, China) to simulate drought and salt stress, respectively, and roots were collected at 48 h. Following the sample process, all plant components were instantly frozen in liquid nitrogen immediately after sampling and kept in a medical refrigerator at -80°C for additional analysis.

### 2.2 Identification of putative *bZIP* genes in *P. grandiflorus*

The complete genome sequence and annotated file of *P. grandiflorus* can be downloaded from the Figshare database (https://doi.org/10.6084/m9.figshare.21511020). TBtools software was used to identify every member of the bZIP TFs in the genome of *P. grandiflorus* (Chen et al., 2020). TBtools software was used to extract all genes sequences in the *P. grandiflorus* genome. The hidden Markov model file in the *bZIP* domain was used as a template to identify each gene and obtain the candidate PgbZIP protein sequence. For validation, the candidate sequences were uploaded to the NCBI CD-search tools and the plant transcription factor database. After removing redundant and structurally incomplete domain sequences, the final PgbZIP protein was obtained. Use the web tool ExPASy ProtParam (https://web.expasy.org/protparam/) to search the molecular weight (MW), isoelectric point, grand average of hydropathicity (GRAVY) of PgbZIP proteins.

### 2.3 Chromosomal location and gene duplication analysis of *PgbZIP* genes

The genome annotation file of *P. grandiflorus* was analyzed and the chromosome location information of *PgbZIP* gene was obtained, and its corresponding chromosomal physical location was mapped using Tbtools software. The genomic information of *Malus domestica* (GCF_002114115.1), *Cannabis sativa* (GCA_900626175.1), *Vitis vinifera* (GCF_000003745.3), *Solanum lycopersicum* (GCF_000188115.4) and *Oryza_sativa* (GCA_001433935.1) were downloaded from the NCBI database. MCScanX software was used to analyze the gene duplication and collinearity of *PgbZIP* gene family (Wang et al., 2012). The *PgbZIP* gene replication events were visualized using the TBtools software’s “Advanced Circos” function, and the collinearity relationship between *P. grandiflorus* and other species was shown using the “Dual Systeny Plot” function.

### 2.4 Phylogenetic analyses of the PgbZIP and AtbZIP proteins

The TAIR database (https://www.arabidopsis.org/) provided the sequence of the *A. thaliana* bZIP protein. MEGA X was used to perform multiple sequence alignment of AtbZIP, IibZIP (Jiang et al., 2022) and PgbZIP protein, respectively (Kumar et al., 2018). The phylogenetic tree was constructed by neighbor- joining (NJ) method, and the bootstrap was repeated 1000 times. PgbZIP proteins are divided into different subfamilies based on *A. thaliana* bZIP proteins (Jakoby et al., 2002).

### 2.5 Analyses of gene structure and cis-element compositions of *PgbZIP*

The *PgbZIP* gene family was subjected to motif analysis using the Multiple Em for Motif Elicitation website. The parameters were set to Classic mode, and ten motifs were discovered (Bailey et al., 2009). Extract the upstream 2 kb sequence of the *PgbZIP* family gene using the “GXF Sequences Extract” tool in TBtools software, and then identify and retrieve the *cis*-elements of the gene family through the PlantCare database (http://bioinformatics.psb.ugent.be/webtools/plantcare/html/) for analysis and comparison (Lescot et al., 2002).

### 2.6 Analysis of the expression profile of the *PgbZIP* gene family in different tissues

The NCBI Sequence Read Archive (SRA) database has the raw transcriptome data for the eight *P. grandiflorus* tissues that were used in this investigation. These accession numbers are as follows: SER912510-SER912517 (Kim et al., 2020). The expression of every *PgbZIP* gene used to be quantified in fragments per kilobase of exon model per million mapped fragments (FPKM). All FPKM values have been processed using row scale transformation, and heatmaps were generated using TBtools.

### 2.7 RNA extraction and evaluation of *bZIP* gene expression patterns in *P. grandiflorus*

Total RNA was extracted from leaves, roots and stems of 1-year-old *P. grandiflorus*, drought, and salt stress treated *P. grandiflorus* seedlings, respectively, using the RNAprep Pure Plant Kit (TIANGEN Biotech Co.,Ltd, Beijing, China). Then, The first strand cDNA was synthesized using MS 1st Strand cDNA Synthesis SuperMix for qPCR (+gDNA wiper) kit (Msunflowers Biotech Co.,Ltd, Beijing, China) reverse transcribed RNA as a template, and finally, qRT-PCR experiments were performed using 2×SYBR Green qPCR Mix (With ROX) kit (Shandong SparkJade Biotechnology Co., Ltd, Shandong, China) kit. qRT-PCR experiments had been carried out the use of an AriaMx real-time PCR system (Agilent Technologies, USA). The *PgGAPDH* had been used as a reference gene (Ma et al., 2016), and three technical replicates had been set up for each experiment. The relative expression of genes was computed by the 2^− ΔΔCT^ Method (Livak and Schmittgen, 2001). The Primer3web (version 4.1.0) website (https://primer3.ut.ee/) was used to layout unique primers (Untergasser et al., 2012). GraphPad Prism (v8.0.2) software program was once used to draw the histogram of relative expression of genes, which was once analyzed by *t*-test.

### 2.8 Statistical analysis

PlantTFDB was used to identify TFs for the whole genome proteins of *P. grandiflorus*, and the online database of Pfam was used to annotate the conserved domains of genes. The correlation between transcriptomes was calculated using a Python script primarily based on the Pearson correlation coefficient (PCC) (Zhang et al., 2017). Visualization of correlation network diagrams using Cytoscape (v3.7.1) software (Shannon et al., 2003). The GO (Gene Ontology) and KEGG (Kyoto Encyclopedia of Genes and Genomes) enrichment analysis using the R language package clusterProfiler (Zhang et al., 2017).

## 3 Results

### 3.1 Identification and physicochemical properties of the *bZIP* genes in *P.grandiflorus*

After removing redundant and incomplete domain sequences, we finally identified 47 *bZIP* genes in *P.grandiflorus* (**Supplementary Table 1**). In subsequent analyses, we named the genes *PgbZIP1* to *PgbZIP47* based on their location on the chromosome or contig (from Pg_chr01_03120T to Pg_contig01856_00120T). Then, we analyzed the physicochemical parameters of the PgbZIP protein in detail. The results confirmed that the amino acid (AA) size of the PgbZIP proteins ranged from 132 AA (PgbZIP40) to 704 AA (PgbZIP13). The molecular weight (MW) of the PgbZIP proteins ranged from 14840.52 Da to 75818.79 Da, with an theoretical isoelectric point (pI) of 4.66 (PgbZIP28) to 9.77 (PgbZIP27). The GRAVY values of all PgbZIP proteins were less than 0, suggesting that all PgbZIP proteins may be hydrophilic.

### 3.2 Chromosome location and replication events of *bZIP* genes in *P.grandiflorus*

**Figure 1:**
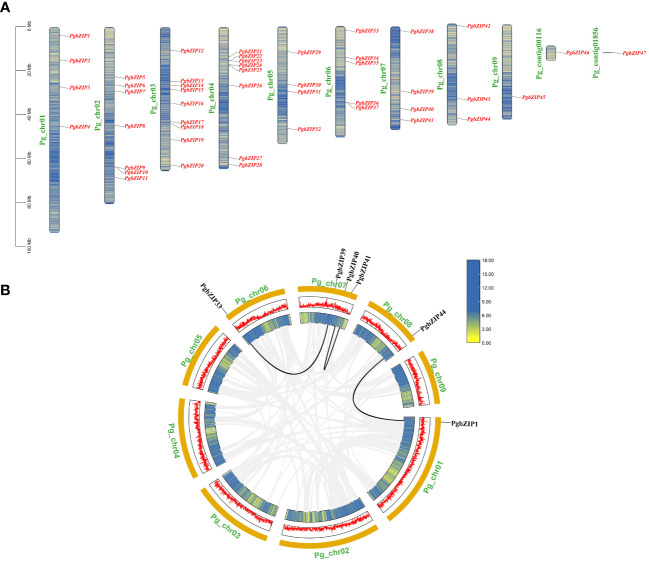
The distribution information of *PgbZIP* genes on chromosomes and gene replication events in the *P.grandiflorus*. **(A)** Information on the location of the *PgbZIP* genes on the *P.grandiflorus*. **(B)** Gene duplication events of *bZIP* genes in *P.grandiflorus*. Black lines indicate gene segmental duplication events in the *PgbZIP* gene, and gray lines show that collinear pairs of all *P.grandiflorus* genes. The yellow lines and red bar indicate the genes density in each chromosome.

The distribution of *PgbZIP* genes on the chromosomes of *P.grandiflorus* did not show any obvious pattern, with 47 *PgbZIP* genes unevenly distributed on 9 chromosomes and 2 contigs (**Figure 1**). Chromosome 3 contained the most *bZIP* genes (9), and chromosome 9, Pg_contig00116 and Pg_contig01856 contained the fewest *bZIP* genes (only a single gene each). The *bZIP* genes were distributed on all chromosomes, but none of the genes showed a preferential distribution on a particular chromosome.

Investigations into the gene replication events in *P. grandiflorus* were conducted to understand the expansion of the *PgbZIP* genes. Gene duplication within the same chromosome or on different chromosomes, but without one following the other, is considered a segmental duplication event. In the *PgbZIP* gene family, four *PgbZIP* gene pairs were generated by segmental duplication. It is worth noting that 1 pair of segmental duplication events (*PgbZIP5* and *PgbZIP46*) is not shown in the **Figure 1B**, because the *PgbZIP46* gene was not mapped to the chromosome and was distributed on the contig.

### 3.3 Phylogenetic analysis of the *PgbZIP* gene family

**Figure 2:**
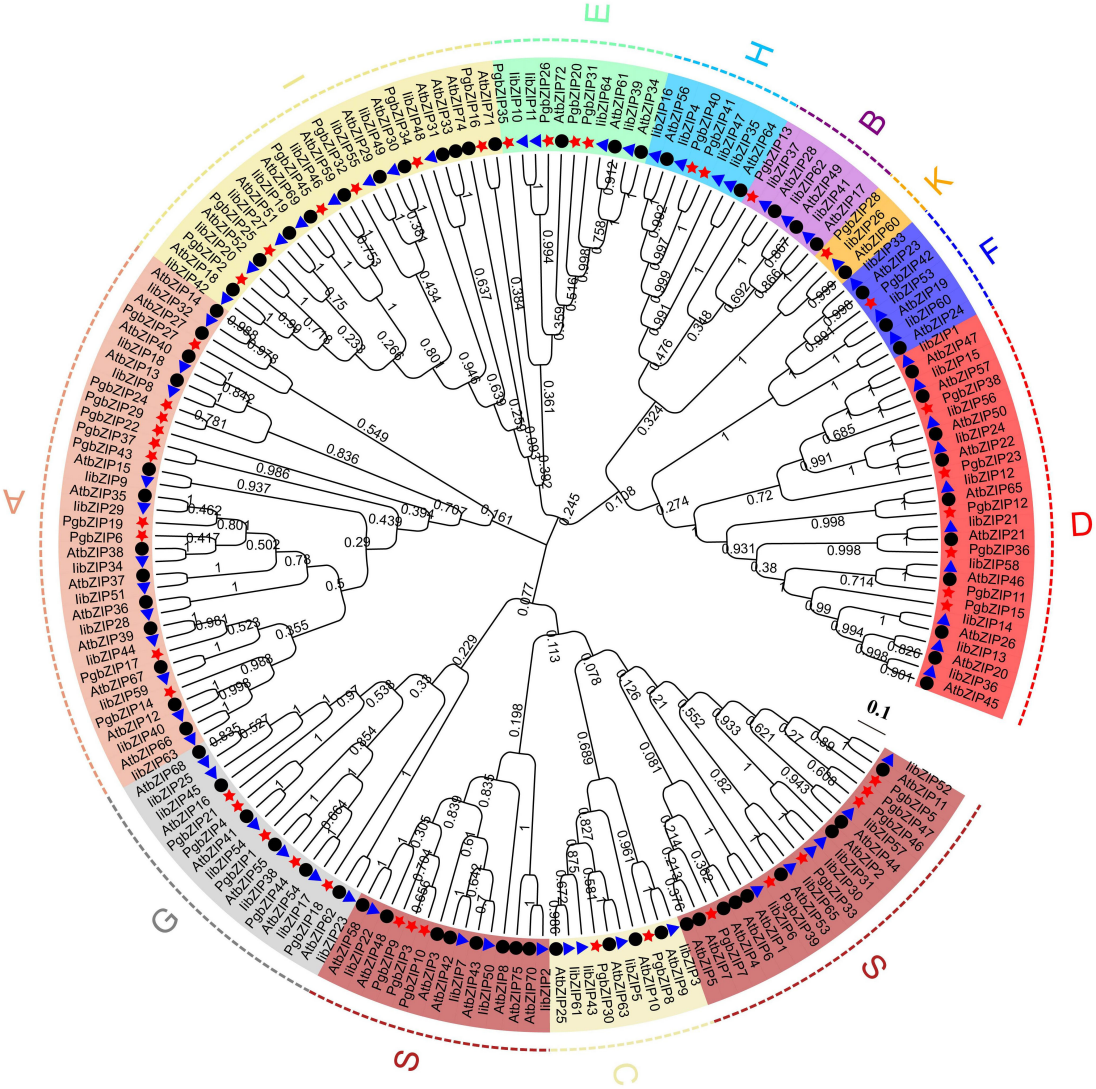
The phylogenetic relationship and classification of the of the bZIP proteins in *P. grandiflorus*, *I. indigotica* and *A. thaliana*. The bootstrap value were set to 1000 replications. Subfamilies are marked with distinct colors. The red star, blue triangle and black dots point out the bZIP proteins of *P. grandiflorus, I. indigotica and A. thaliana*, respectively.

To explore the evolutionary relationships and classification of the *PgbZIP* gene family, we constructed NJ phylogenetic trees using MEGA X software (**Supplementary Table 2**). Based on the earlier classification results of the *A. thaliana* and *I. indigotica* bZIP gene family, 47 PgbZIP proteins were divided into 11 subfamilies (**Figure 2**), comprising A (10), B (1), C (2), D (6), E (4), F (1), G (5), H (2), I (6), K (1) and S (9). The outcome of phylogenetic tree evaluation confirmed that the *bZIP* genes of *P. grandiflorus*, *A. thaliana* and *I. indigotica* were highly homologous in each cluster.

### 3.4 Gene structure, motif and *cis*-element analysis of *PgbZIP*

**Figure 3:**
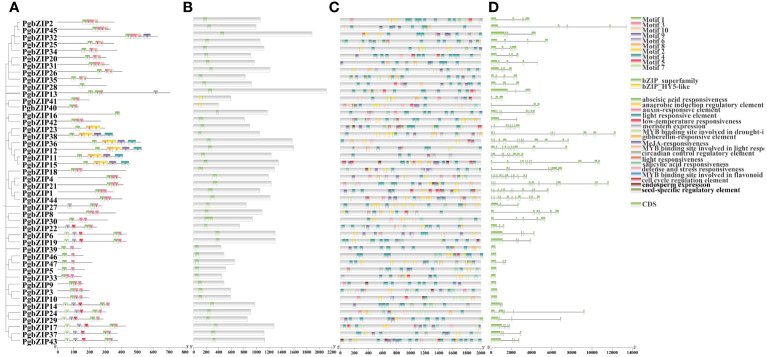
Phylogenetic analysis motifs, conserved domains and *cis*-element analysis of the *PgbZIP* genes. **(A)** Distribution of motifs within each PgbZIP protein. **(B)** Conserved domains of PgbZIP proteins. **(C)** Distribution of different types of *cis*-elements in the promoter region of the *PgbZIP* genes. **(D)** *PgbZIP* gene structures, lines indicate introns. **(D)** Distribution of different types of *cis*-elements in the promoter region of the *PgbZIP* genes.

To explore the structural similarity of PgbZIP proteins, 10 conserved motifs have been recognized the use of the MEME website (**Figure 3A**). These motifs vary in size from 21–50 AA, with at least 1 to 5 motifs distributed across 47 PgbZIP proteins, and all sequences have motif 1. Members of gene families with close evolutionary relationships have similar motif compositions; for example, G subfamily members all have motifs 1 and 3. Furthermore, all PgbZIP proteins contain conserved domains of the *bZIP* genes, proving that the outcome of gene identification had been dependable (**Figure 3B**).

To research the potential transcriptional regulatory role of *PgbZIP* genes, in this study, *cis*-elements were extracted from the PlantCARE database using 2K bp upstream of the *PgbZIP* genes as the promoter region. A total of 929 valuable *cis*-elements were recognized in the promoter regions of 47 *PgbZIP* genes, these *cis*-elements could be broadly categorized into three groups (**Figure 3C**) (**Supplementary Table 3**). Relevant to plant growth include light responsive elements, meristem expression, endosperm expression, anaerobically induced regulatory elements, seed-specific regulatory response elements, cell cycle regulatory elements, and circadian regulatory elements. Hormone response related elements include MeJA responsive elements, auxin responsive elements, abscisic acid responsive elements, and gibberellin responsive elements. Involved in abiotic stress related including stress responsive and defense elements and low-temperature responsive elements. Interestingly, the *PgbZIP* gene has *cis*-elements that bind to the *MYB* gene, participating in photoresponse, drought response, and flavonoid synthesis. This suggests that the *PgMYB* gene may regulate *PgbZIP*, forming regulatory networks that exercise different biological functions.

In order to further clarify the evolution of the *PgbZIP* genes, we compared the *PgbZIP* gene sequences and analyzed the coding regions and introns (**Figure 3D**). Except for seven members of the S family who do not have introns, the number of introns in other members is distributed between 1 and 12. As anticipated, members of the same subfamily have a relatively conserved number of introns, such as the H subfamily, which has 2 introns, and the E subfamily, which has 3 to 4 introns.

### 3.5 Syntenic analysis of *PgbZIP* genes with other species

**Figure 4:**
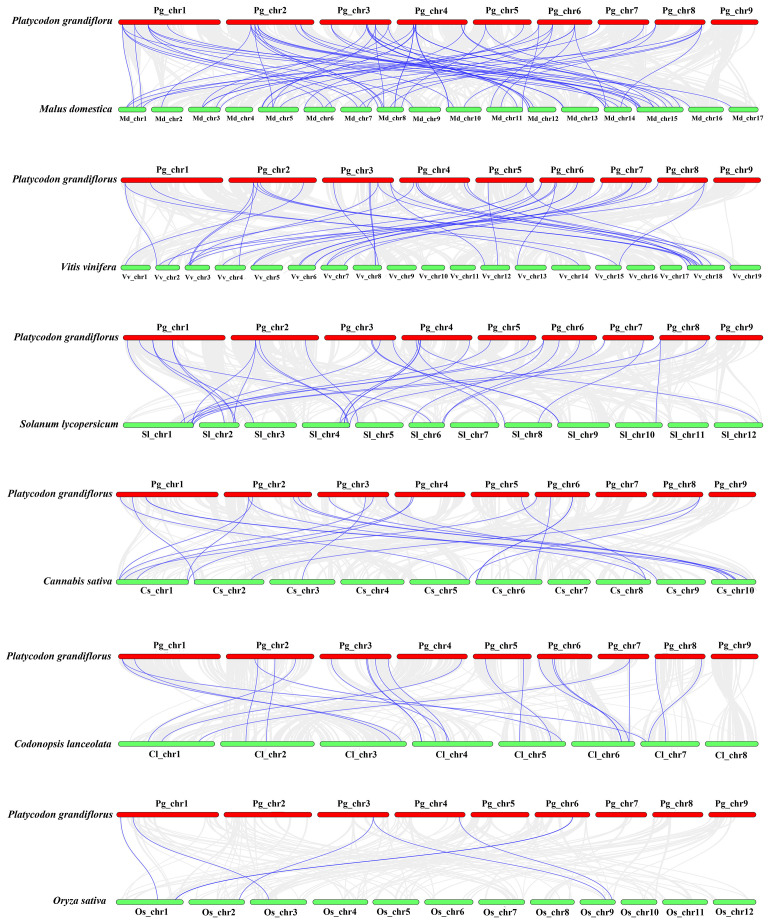
Synteny analysis of *bZIP* genes between *P.grandiflorus* and *M. domestica*, *V. vinifera*, *S. lycopersicum, C. sativa, C. lanceolata*, and *O. sativa.* Gray lines symbolize the colinear blocks within *P.grandiflorus and* other genomes, Purple lines represent the syntenic *bZIP* gene pairs.

For investigating further the relationship of *bZIP* genes evolution among various plants, we compared interspecific synteny of *P. grandiflorus* with that of five dicotyledonous plants and one monocotyledonous plant (*O. sativa*) (**Figure 4**). Dicotyledonous plants include three fruit (*M. domestica*, *V. vinifera* and *S. lycopersicum*), and two medicinal plants (*C. sativa* and *C. lanceolata*). The results showed that the collinearity relationship between *PgbZIP* genes and *MdbZIP* genes was the closest, with 79 pairs of genes, 40 pairs of *PgbZIP* and *VvbZIP* genes, 32 pairs of *PgbZIP* and *SlbZIP* genes, 23 pairs of *PgbZIP* and *ClbZIP* genes, and the same pairs of *CsbZIP* genes. *PgbZIP* and *OsbZIP* had the worst collinearity relationship, with only 7 pairs of genes. These results indicate that most of these homologous *bZIP* genes occur after the differentiation of dicotyledons and monocotyledons. It is worth noting that *PgbZIP1* (*Pg_chr01_03120T*) and *PgbZIP19* (*Pg_chr03_29870T*) have syntenic gene pairs with 6 other species, which may have existed before species divergence and participated in the evolution of these plants.

### 3.6 Divergent expression of the *bZIP* gene in *P. grandiflorus* tissues and verification of candidate genes by quantitative real-time PCR

**Figure 5:**
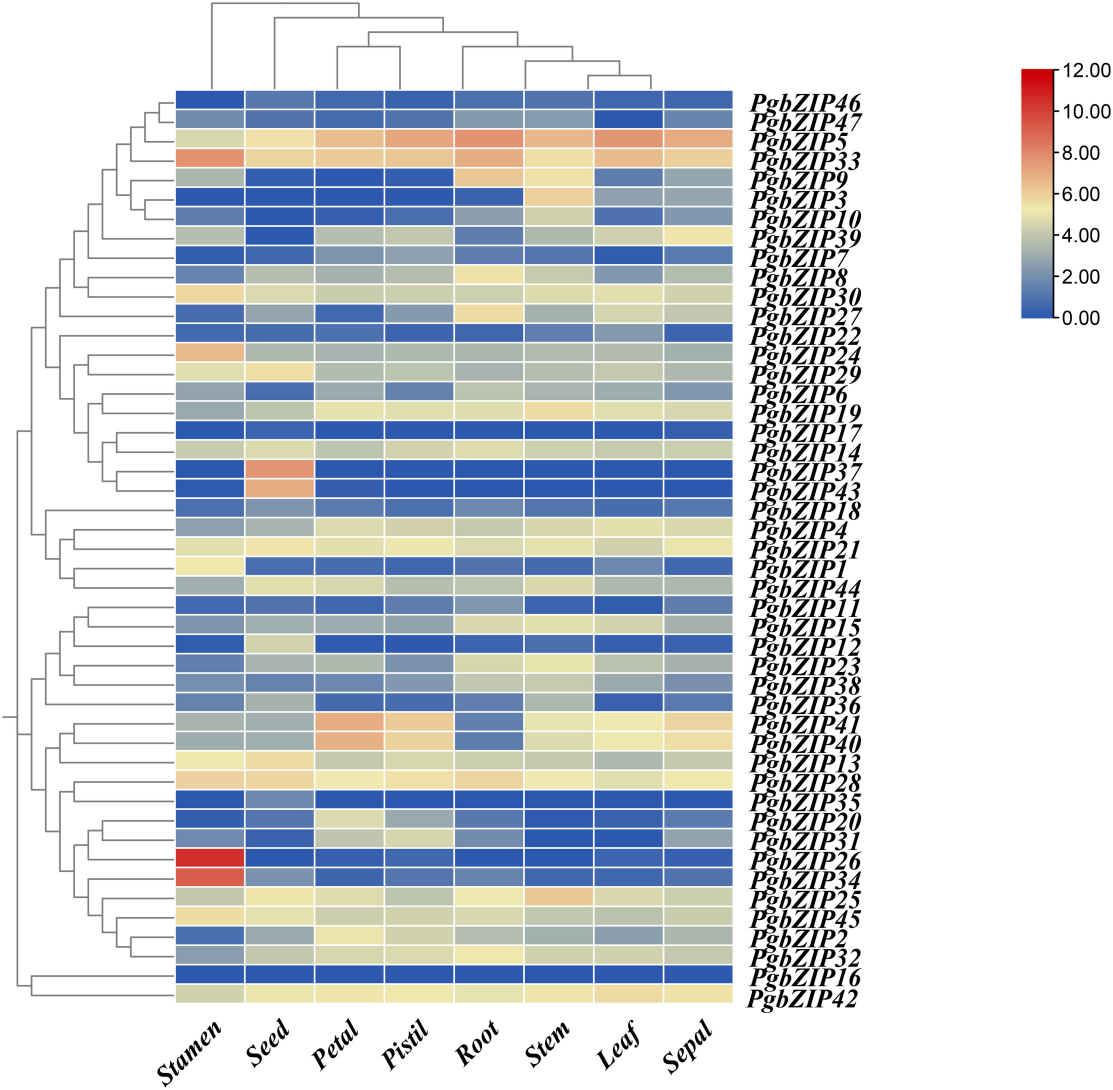
Heatmap of *bZIP* gene expression profiles in eight tissues of *P. grandifloru*. Data represent FPKM values from transcriptome data come from 8 tissues, including seed, leaf, petal, pistil, root, sepal, stamen and stem. Gene expression using Log_2_(FPKM+1) logarithmic transformation treatment.

To investigate the expression profile of *bZIP* genes in *P. grandiflorus* tissues, the gene relative expression of 47 *PgbZIP* genes were analyzed based on RNA-seq data of *P. grandiflorus* root, leaf, seed, petal, stem, stamen, pistil, and sepal (**Figure 5**) (**Supplementary Table 5**). The findings indicated that 20 genes were expressed in leaf, 28 genes in petal, 34 genes in pistil, 32 genes in root, 38 genes in seed, 37 genes in sepal, 37 genes in stamen and 37 genes in stem (FPKM>0.5). A total of 10 genes (*PgbZIP5*, *PgbZIP13*, *PgbZIP14*, *PgbZIP21*, *PgbZIP25*, *PgbZIP28*, *PgbZIP30*, *PgbZIP33*, *PgbZIP42* and *PgbZIP45*) were highly expressed in all tissues (FPKM>10), and these genes probably participating in the whole development processes of *P. grandiflorus*. *PgbZIP16* and *PgbZIP17* were not expressed in all tissues and may be pseudogenes or require specific conditions to activate expression. Interestingly, *PgbZIP* genes are also tissue-specific. For example, *PgbZIP26* and *PgbZIP34* are expressed only in stamens. *PgbZIP12*, *PgbZIP35*, *PgbZIP37* and *PgbZIP43* are only expressed in seeds. These genes involved in specific tissue expression may only be involved in the biological process of this tissue.

**Figure 6:**
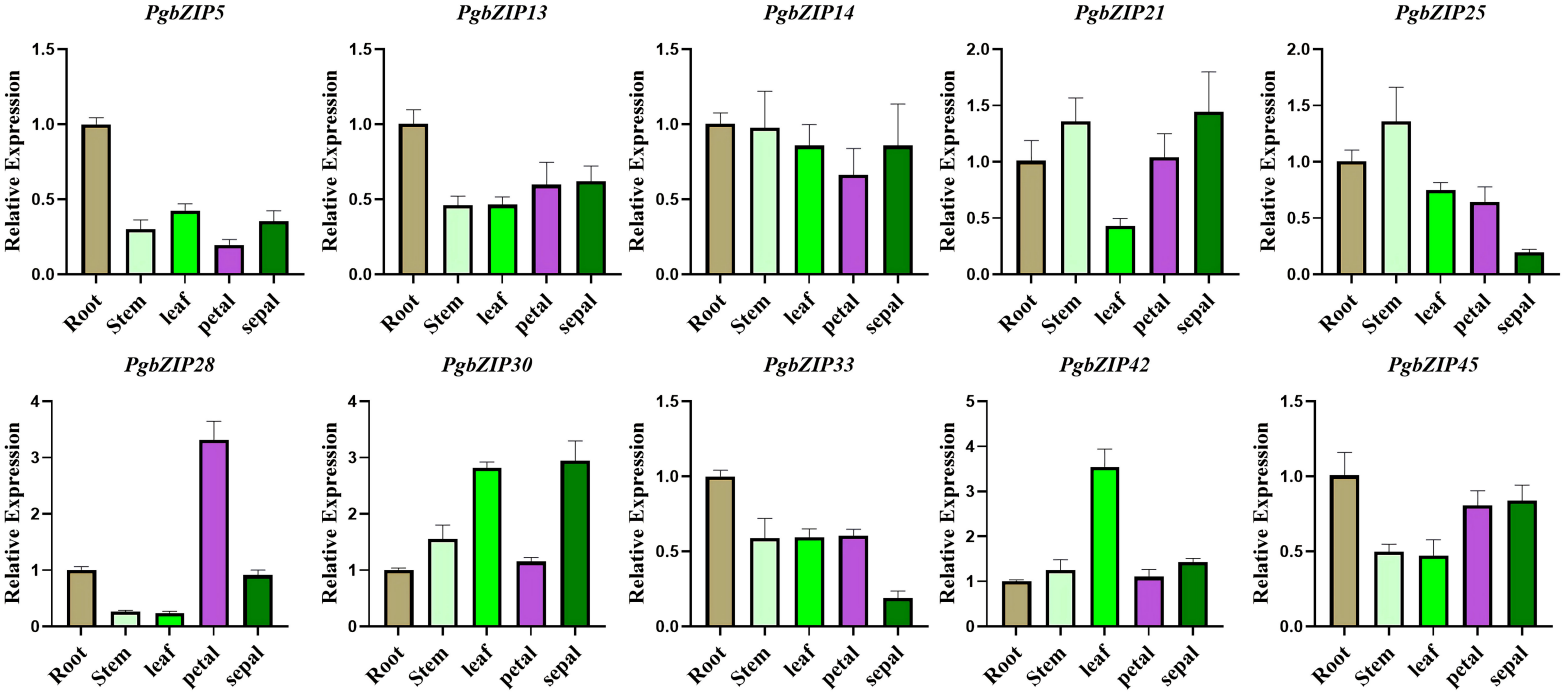
qRT–PCR analysis of *PgbZIP* genes in five different tissues of *P. grandiflorus*. Different colors represent different tissues. Use of the *PgGAPDH* gene as a reference gene.

As mentioned above, subfamilies A (*PgbZIP14*), B (*PgbZIP13*), C (*PgbZIP30*), F (*PgbZIP42*), G (*PgbZIP21*), I (*PgbZIP25/45*), K (*PgbZIP28*) and S (*PgbZIP5/33*) have been expressed at very high levels in various tissue parts of *P. grandiflorus*, and are probably participated in the regulation of developmental process in all tissues. Therefore, those 10 *PgbZIP* genes were selected as candidate genes for qRT-PCR experiments to validate in this research (**Supplementary Table 6**). The relative expression of the 10 candidate genes were basically consistent to the expression trends obtained from the RNA-seq data (**Figure 6**). Notably, *PgbZIP28* showed higher expression in petals, and *PgbZIP30* and *PgbZIP33* were expressed at higher levels in sepals than stems. All of the above suggest that these 10 *PgbZIP* genes possibly are closely involved to the developmental process of *P. grandiflorus*.

### 3.7 Analysis of expression profile of *PgbZIP* genes family under drought and salt stress

**Figure 7:**
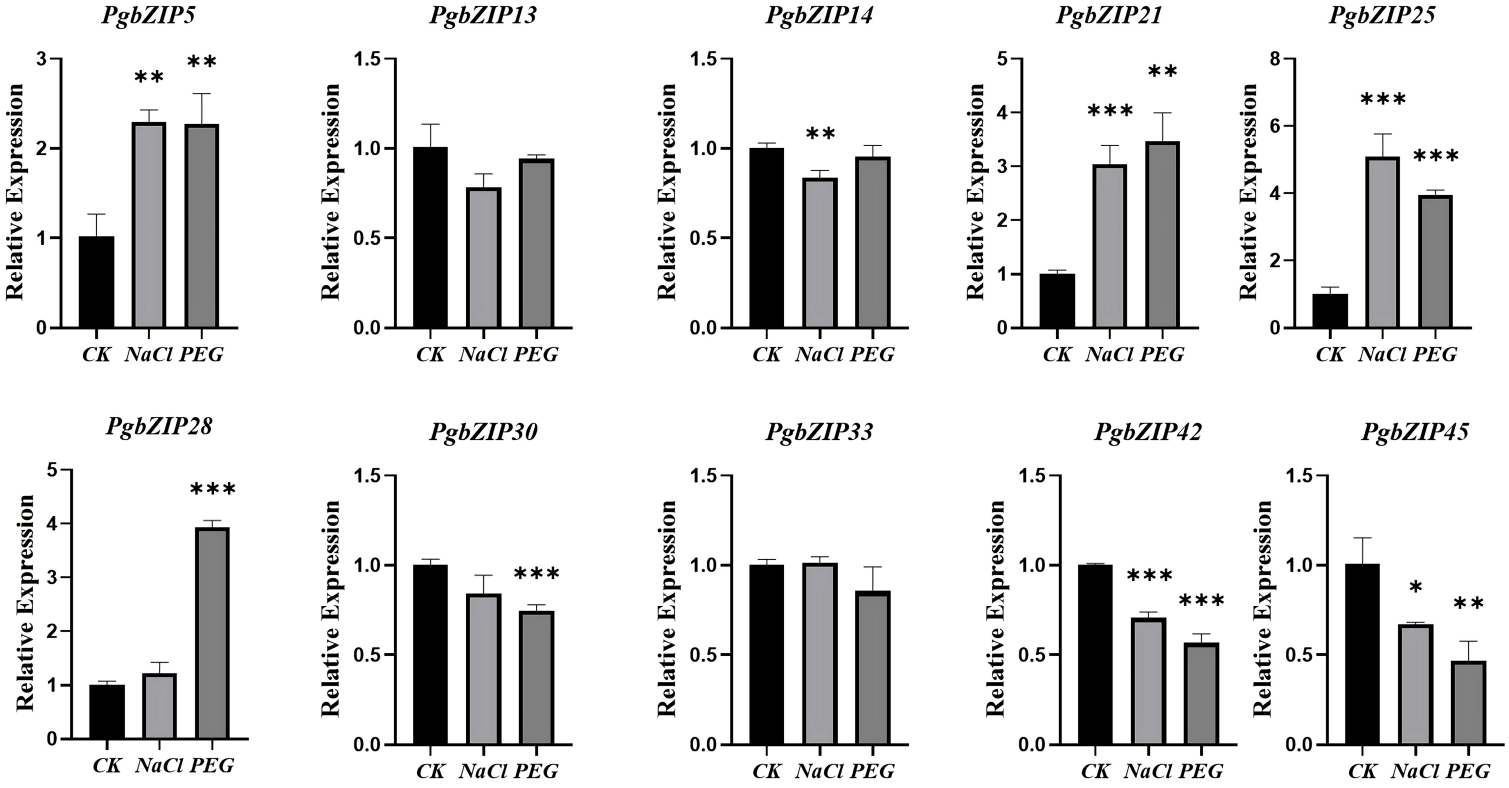
qRT-PCR analysis of *PgbZIP* genes under drought and salt stress. CK: normal growth group; Salt stress and drought stress simulated using NaCl and PEG. The *PgGAPDH* gene was used as a reference gene. The significance analysis was carried out the use of a t-test. *, **, *** indicate significant difference in *p* < 0.05, *p* < 0.01, and *p* < 0.001.

The root of *P. grandiflorus* is the main medicinal part. For studying the potential role of *PgbZIP* gene family under abiotic stress, the qRT-PCR experiments analysis was conducted using the roots of *P. grandiflorus* seedlings under drought and salt stress as templates (**Figure 7**). Compared with the normally growth group (CK), the expression levels of 4 genes (*PgbZIP5*, *PgbZIP21*, *PgbZIP25* and *PgbZIP28*) were increased under both drought and salt stress. *PgbZIP33* was only highly expressed under salt stress, but its expression was reduced under drought stress. These *PgbZIP* genes with increased expression levels under drought and salt stress could help *P. grandifloru* to resist abiotic stress.

### 3.8 Correlation network and enrichment analysis of *PgbZIP* genes

**Figure 8:**
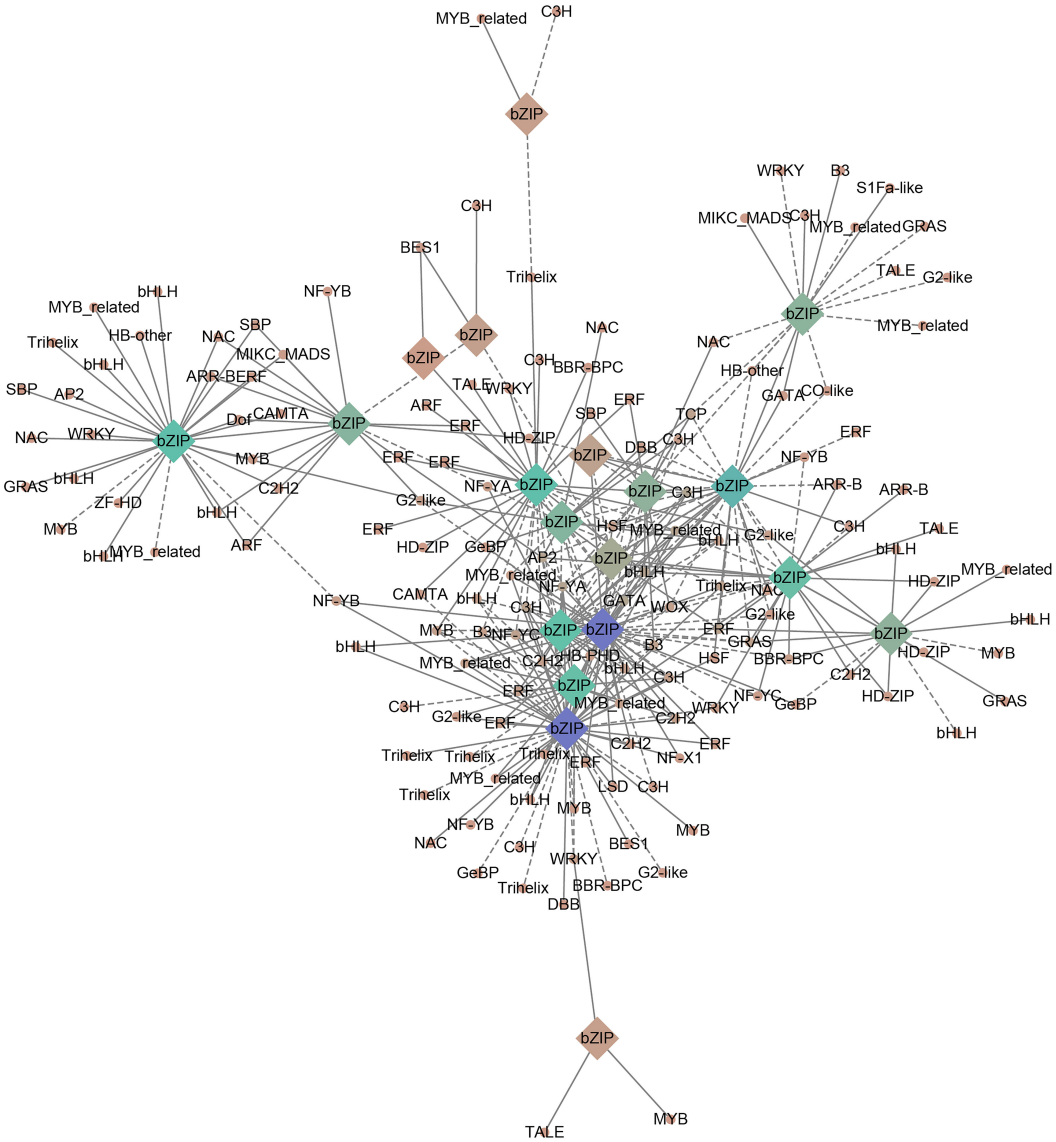
*PgbZIP* genes correlation network established using RNA-seq data from 8 tissues of *P.grandiflorus*. Circles represent co-expressed transcription factors, and diamonds represent the *PgbZIP* genes. Positive and negative correlations are indicated by solid and dashed lines, respectively. The shade of the color indicates the amount of genes correlated.

The *bZIP* genes often form an interaction networks with various TFs to participate in plant developmental processes. In order to mine the transcriptional regulatory network of *PgbZIP* in different tissues, this study characterized TFs in the RNA-seq data of 8 different tissues of *P. grandiflorus* and constructed a correlation network. A total of 1567 TFs were identified and categorized into 58 gene families, among which the top 5 were ERF (136), bHLH (118), C2H2 (115), MYB (105) and NAC (90). The expression profiles of all genes in the RNA-seq data of 8 different tissues of *P.grandiflorus* were analyzed using Python script to explore the TFs co-expressed with the *PgbZIP* gene. In the correlation network, a total of 19 *PgbZIPs* were co-expressed with 149 TFs, and 168 nodes with 361 network pairs were found. The positive (r > 0.7) (P < 0.05) and negative (r < -0.7) (P < 0.05) correlation network pairs are 232 and 129, respectively (**Figure 8**). *PgbZIP45*, *PgbZIP30*, *PgbZIP13* and *PgbZIP32* were presented as hub genes (degree≧30). The highest correlations (degree≧7) among TFs were Pg_chr05_09390T (GATA), Pg_chr08_31480T (C3H), Pg_chr02_03150T (MYB-related), Pg_chr01_14460T (AP2), Pg_chr06_07740T (bHLH), and Pg_contig26001_00320T (NF-YA) (**Supplementary Table 7**). These TFs has been shown to function in regulating plant growth and development in a large number of studies. Therefore, *PgbZIP* genes could be interacting with these TFs to form a regulatory network and participate in the development process of *P.grandiflorus*.

**Figure 9:**
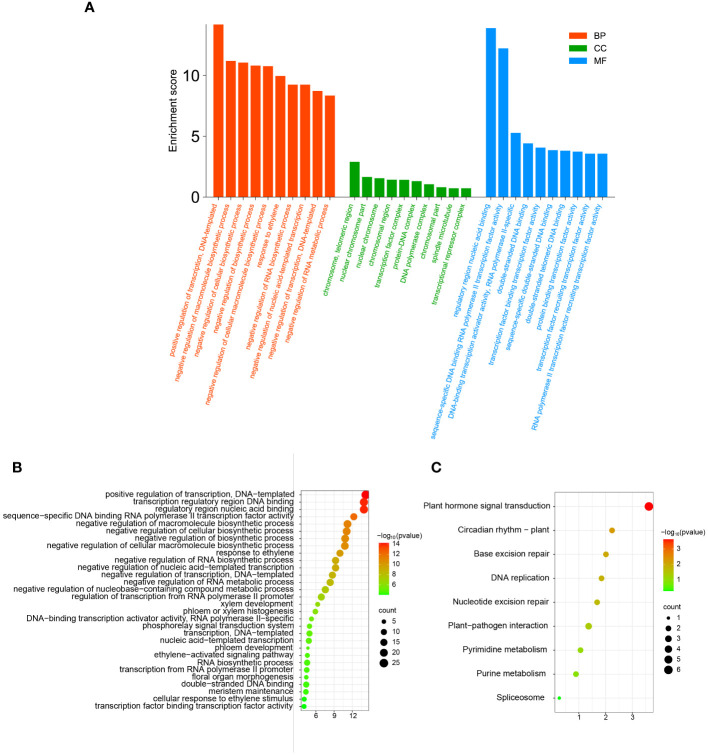
The GO annotation and KEGG pathway enrichment analysis of TFs in correlation networks. **(A)** The GO annotation of TFs in correlation networks. **(B)** The GO enrichment analysis of TFs in correlation networks. **(C)** The KEGG pathway enrichment analysis of TFs in correlation networks.

In order to further clarify the functions of TFs in the network in biological processes and the pathways involved, we performed GO annotation, GO and KEGG enrichment analyses of all TFs in the correlation network (**Figure 9**). The results of the analysis of the GO annotation findings suggests that those genes function in the molecular function classification for recruitment of TFs and activation of transcriptional activity (**Figure 9A**). In the cellular component classification, these genes were found to be predominantly distributed in the telomeric region, chromosome, nuclear chromosome and spindle microtubule. In biological processes, these genes are mainly involved in response to ethylene, negative regulation of macromolecular biosynthesis processes, negative regulation of RNA metabolic processes and ethylene response (**Supplementary Table 8**).

Similarly, we performed GO and KEGG enrichment analysis of TFs in the correlation network. The outcome of GO enrichment analysis indicated that the main functions of these genes are transcription factor binding transcription factor activity, cellular response to ethylene stimulus, meristem maintenance, response to ethylene, xylem development, phloem development, floral organ morphogenesis, meristem maintenance, and so on (**Figure 9B**). The outcome of KEGG enrichment indicated that these TFs were primarily enriched in circadian rhythm, phytohormone signal transduction, nucleotide excision repair, plant-pathogen interaction, pyrimidine metabolism, purine metabolism and so on (**Figure 9C**). These outcomes indicate that the TFs in the correlation network are participating in most bioprocesses in plants and play a certain role.

## 4 Discussion

The bZIP transcription factors are among the most widely distributed and conserved families of eukaryotic TFs, and have been more thoroughly researched in the plant field (Li et al., 2016). The bZIP TFs are widely involved in plant developmental process, biotic and abiotic stress response, and regulation of plant secondary metabolite synthesis such as terpenoids (Zhong et al., 2021). *P. grandiflorus* is a species of both medicine and food. Its young shoots and roots are often used to make pickles, cold soups and sauces. Similarly, the dried root of *P. grandiflorus* is a traditional Chinese medicine (TCM) called PLATYCODONIS RADIX, which has a variety of pharmacological effects and is a commonly used bulk of TCM (Ma et al., 2021). In the context of the post-epidemic era, the concept of “preventing disease” has gradually taken root in people’s hearts, and the concept of health care based on the same source of medicine and food will be the focus of attention at home and abroad in the future. As one of the representative medicinal materials of the same source of medicine and food, the international requirement for its raw materials is increasing, but the quality of different production areas is different. Therefore, studying the molecular regulatory mechanisms of the developmental process of *P. grandiflorus* to increase its productivity and high quality has emerged as a research hotspot. The bZIP TFs were extensively studied in various plants, but not in *P. grandiflorus*. In this present work, 47 *PgbZIP* genes were recognized by bioinformatics methods, which was similar to most previous reports, such as 49 in *Solanum tuberosum* (Wang et al., 2021; Wang et al., 2021), 54 in *Litchi chinensis* (Hou et al., 2022), 59 in *Castanea mollissima* (Zhang et al., 2023), 65 in *Lagenaria siceraria* (Wang et al., 2022) and 65 in *I. tinctoria*. A deeper analysis revealed that all *PgbZIP* genes included the bZIP conserved domains, suggesting that the identification results were reliable and accurate. Similarly, motif1 and motif3 comprise a leucine zipper region and base region of the *bZIP* genes, consistent with the identification of bZIP TFs in *Nicotiana tabacum* (Duan et al., 2022).

Phylogenetic analysis can identify homologous genes in different species for the purpose of predicting the function of unknown genes. In this study, a total of 47 PgbZIP protein and 75 A*. thaliana* bZIP protein have been employed to build a phylogenetic trees, which was divided into 11 subfamilies and showed good clustering results. In subfamily A, the PgbZIP6 and PgbZIP19 proteins were homologous with AtbZIP36, and the PgbZIP17 protein was homologous with AtbZIP36, indicating that the three PgbZIP proteins probably have important functions in ABA induction and stress treatment (Choi et al., 2000). Similarly, in subfamily D, AtbZIP46 is participating in the progression of *A. thaliana* flower and plays a role in regulating the size of meristems or floral organ number (Chuang et al., 1999), and its homologous gene *PgbZIP15* probably has the same functionality. Notably, the H subfamily have two genes, namely, *HY5* (*AtbZIP56*) and *HYH* (*AtbZIP64*), while the H subfamily in the *PgbZIP* gene family contains only two *HY5* genes (*PgbZIP40*, *PgbZIP41*) but no *HYH* gene, suggests that there could be possible gene expansions and deletions in the *PgbZIP* gene family that may have occurred during the evolutionary process.

The function of transcription factors is highly correlated with their expression patterns (Wang et al., 2023). Earlier research has indicated that the *bZIP* gens were participating in the developmental processes of various tissues and organs in plants. Coexpression of AtbZIP10/25 with ABI3 significantly increases the activation capacity of the At2S1 promoter to form a regulatory complex for seed-specific expression (Lara et al., 2003). The *AtbZIP9* gene is involved in regulating leaves and vascular bundle development (Silveira et al., 2007). Overexpression of *OsbZIP49* in *O. sativa* reduced internode length and plant height in transgenic rice, which exhibited a tiller-spreading phenotype (Ding et al., 2021). Overexpression of the *Capsicum annuum CabZIP1* gene in *A. thaliana* can slow plant growth and decrease the amount of petals (Lee et al., 2006). To identify the regulatory functions of *PgbZIP* genes in *P. grandiflorus* development, 10 *PgbZIP* genes (*PgbZIP5, PgbZIP13, PgbZIP14, PgbZIP21, PgbZIP25, PgbZIP28, PgbZIP30, PgbZIP33, PgbZIP42* and *PgbZIP45*) that were highly expressed in the transcriptome data of 8 tissues were selected as potential genes, the relative expression of those genes in stems, leaves, roots, petals and sepals have been analyzed by qRT-PCR. The relative expression of candidate genes were in accordance with the trend of RNA-seq data, suggesting these 10 candidate genes were participated in the development of various tissues of *P. grandiflorus*. Similarly, the *PgbZIP* genes also showed tissue-specific expression in different organs. Transcriptome data showed that *PgbZIP12*, *PgbZIP35*, *PgbZIP37* and *PgbZIP43* were only expressed in seed species, which predicted that they could be participated in seed developmental processes. A comparable tissue-specific expression patterns have been found for the bZIP TFs in species such as *M. domestica* (Wang et al., 2021; Wang et al., 2021), *Musa nana* (Hu et al., 2016), and *Citrullus lanatus* (Yang et al., 2019).

In recent years, the biosynthesis of natural products has become increasingly popular. However, the molecular mechanisms by which TFs are involved in regulating the biosynthesis of plant secondary metabolism are complex. The triterpenoid saponins of *P. grandiflorus* are very important secondary metabolites, that are mainly enriched in roots (Zhang et al., 2015; Zhang et al., 2015). Root development is a complex process regulated by the expression of multiple genes and influenced by endogenous hormone levels and natural resources (Siqueira et al., 2022). During energy deprivation in *A. thaliana*, *AtbZIP63* activates *AtARF19* expression in response to basal lateral root initiation (Muralidhara et al., 2021). Under stress and normal conditions, different levels of ABA (abscisic acid) regulate the development of plant root architecture, including the initiation and elongation of main, adventitious, adventitious roots, and root hairs, as well as root system hydrophilicity and geotropism (Teng et al., 2023). Similarly, bZIP TFs perform important functions in the control of terpenoids; for example, the bZIP TF AaTGA6 in *A. annua* is involved in the regulation of salicylic acid (SA) in the synthesis of artemisinin (Lv et al., 2019), and AabZIP1 is involved in ABA signaling, which in turn regulates artemisinin biosynthesis (Zhang et al., 2022; Zhang et al., 2022). In this study, *cis*-elements for physiological control, hormonal response and stress response were detected in the promoter region of the *PgbZIP* gene, suggesting that the *PgbZIP* gene with these elements performs an essential function in the regulation of root developmental processes and terpene biosynthesis in *P. grandifloru*.

With the fast speed of modern industrial development and climate change, plants suffer from abiotic stress increasingly frequently in the manner of increase and development, which conduct to a reduction in yield, quality damage, and even plant death (Zhang et al., 2022). To cope with the stress caused by adverse environments, plants have evolved various mechanisms, including the regulation of gene expression through various transcription factors, so that plants can adapt to or escape the effects of stress (Badis et al., 2009). Among these transcription factors, the bZIP TFs has been widely reported to enhance the ability of response to biotic and abiotic stresses. For example, *OsbZIP71* gene in *O. sativa* enhances the tolerance to drought and salinity by activating the expression of the OsNHX1 protein and COR413-TM1 protein (Liu et al., 2014). In *V. vinifera*, the *VvbZIP23* expression is induced by a number of abiotic stresses, including cold, salinity and drought stress (Tak and Mhatre, 2013). The *Hordeum vulgare* bZIP TF HvABI5 is participating in ABA-dependent regulation of resistance to drought stress (Collin et al., 2020). In this study, *PgbZIP5*, *PgbZIP21*, *PgbZIP25* and *PgbZIP28* genes with significantly considerably elevated expression levels beneath drought and salinity stress were verified through qRT-PCR experiments. Additionally, *PgbZIP33* was only highly expressed under salt stress. Among them, *PgbZIP5* is the homologous gene of *AtbZIP11*, which may have the same features in resistance to stress (Weltmeier et al., 2009). *PgbZIP21* and *PgbZIP28* have *cis*-elements participating in defense and stress responses, and *PgbZIP21*, *PgbZIP25*, and *PgbZIP33* have binding sites for drought regulation with *PgMYB* genes. *PgbZIP28* and *PgbZIP33* also contain low-temperature responsiveness elements. These 5 genes also have co-expression links with *bHLH, GATA, MYB* of the stress resistance related genes. It is hypothesized that those TFs might perform key functions in supporting *P. grandiflorus* to resist abiotic stress. However, the specific biological functions of these genes need to be further verified.

## 5 Conclusion

In this work, a number of 47 *PgbZIP* genes were characterized based on genome-wide analysis, and their chromosomal distribution, phylogenetic, motifs, gene structure, *cis*-element prediction, synteny and expression profiles have been comprehensively analyzed. Ten candidate *PgbZIP* genes that are expressed at high levels in all tissues indicate their crucial role in various physiological and biological processes, and the co-express network results also provide evidence for further research. The expression pattern of candidate genes under drought and salt stress provide valuable information for the expression of *PgbZIP* genes under salt and drought stress. These results provide clues to investigate the functions of the *PgbZIP* genes in the developmental processes of different plant organs and abiotic stress responses.

## Data availability statement

This study analyzed publicly available data from the NCBI database (https://www.ncbi.nlm.nih.gov/). Accession numbers: SRR8712510-SRR8712517.

## Author contributions

ZW: Investigation, Software, Writing – original draft, Writing – review & editing. PW: Data curation, Methodology, Writing – original draft. HC: Formal analysis, Software, Writing – original draft. ML: Investigation, Software, Writing – original draft. LK: Investigation, Methodology, Writing – original draft. HW: Resources, Writing – original draft. WR: Resources, Software, Writing – original draft. QF: Formal analysis, Supervision, Validation, Writing – original draft. WM: Funding acquisition, Resources, Visualization, Writing – original draft, Writing – review & editing.
